# Impact of aging on diaphragm muscle function in male and female Fischer 344 rats

**DOI:** 10.14814/phy2.13786

**Published:** 2018-07-06

**Authors:** Obaid U. Khurram, Matthew J. Fogarty, Tiffany L. Sarrafian, Arjun Bhatt, Carlos B. Mantilla, Gary C. Sieck

**Affiliations:** ^1^ Department of Physiology and Biomedical Engineering Mayo Clinic Rochester Minnesota; ^2^ School of Biomedical Sciences The University of Queensland Brisbane Australia; ^3^ Department of Anesthesiology and Perioperative Medicine Mayo Clinic Rochester Minnesota

**Keywords:** Aging, diaphragm muscle, fiber type, Sarcopenia, sex differences, transdiaphragmatic pressure

## Abstract

The diaphragm muscle (DIAm) is the primary inspiratory muscle in mammals and is active during ventilatory behaviors, but it is also involved in higher‐force behaviors such as those necessary for clearing the airway. Our laboratory has previously reported DIAm sarcopenia in rats and mice characterized by DIAm atrophy and a reduction in maximum specific force at 24 months of age. In Fischer 344 rats, these studies were limited to male animals, although in other studies, we noted a more rapid increase in body mass from 6 to 24 months of age in females (~140%) compared to males (~110%). This difference in body weight gain suggests a possible sex difference in the manifestation of sarcopenia. In mice, we previously measured transdiaphragmatic pressure (Pdi) to evaluate in vivo DIAm force generation across a range of motor behaviors, but found no evidence of sex‐related differences. The purpose of this study in Fischer 344 rats was to evaluate if there are sex‐related differences in DIAm sarcopenia, and if such differences translate to a functional impact on Pdi generation across motor behaviors and maximal Pdi (Pdi_max_) elicited by bilateral phrenic nerve stimulation. In both males and females, DIAm sarcopenia was apparent in 24‐month‐old rats with a ~30% reduction in both maximum specific force and the cross‐sectional area of type IIx and/or IIb fibers. Importantly, in both males and females, Pdi generated during ventilatory behaviors was unimpaired by sarcopenia, even during more forceful ventilatory efforts induced via airway occlusion. Although ventilatory behaviors were preserved with aging, there was a ~20% reduction in Pdi_max_, which likely impairs the ability of the DIAm to generate higher‐force expulsive airway clearance behaviors necessary to maintain airway patency.

## Introduction

Sarcopenia, which is defined as the age‐related loss of muscle‐specific force and cross‐sectional area, is observed in the diaphragm muscle (DIAm) (Gosselin et al. 1994; Greising et al. 2013, 2015a,b; Elliott et al. 2016a,c) and may underlie the increased susceptibility to respiratory disease observed clinically among older individuals (Sharma and Goodwin 2006; Lowery et al. 2013). Ventilatory behaviors require activation of ~10–30% of total DIAm force capacity, whereas expulsive behaviors such as coughing and sneezing require much higher DIAm forces (Sieck 1988; Sieck and Fournier 1989; Mantilla et al. 2010, 2014a; Fogarty et al. 2018a). The DIAm comprises different fiber types that vary in mechanical and fatigue properties (Sieck et al. 1989a; Geiger et al. 2000). In the DIAm, sarcopenia selectively affects the cross‐sectional areas of type IIx and/or IIb muscle fibers (Greising et al. 2013, 2015b; Elliott et al. 2016c), which are recruited mainly during higher‐force expulsive behaviors. The selective atrophy of type IIx and/or IIb DIAm fibers with aging may diminish the ability to generate forces necessary to accomplish expulsive behaviors that are essential for maintaining airway patency.

Force generation in the DIAm involves the orderly recruitment of motor units (Sieck et al. 1984; Seven et al. 2014). Based on models of motor unit recruitment, it is likely that slow‐twitch (type S) and fast‐twitch, fatigue‐resistant (FR) motor units are recruited first during sustained low‐force ventilatory behaviors, and fast‐twitch, fatigue intermediate (FInt), and fast‐twitch, fatigable (FF) motor units are recruited later and far less frequently, usually during higher‐force, expulsive motor behaviors (Sieck 1988, 1989; Sieck and Fournier 1989; Mantilla and Sieck 2011; Mantilla et al. 2014a; Fogarty et al. 2018a). Transdiaphragmatic pressure (Pdi) measurements reflect DIAm force generation across a range of motor behaviors in cats (Sieck et al. 1989a), rats (Mantilla et al. 2010; Gill et al. 2015; Khurram et al. 2017), and mice (Greising et al. 2013, 2015b). Maximum Pdi (Pdi_max_) is elicited by bilateral phrenic nerve stimulation and can be used to normalize DIAm force generation across animals during different behaviors (Sieck et al. 1989a; Mantilla et al. 2010; Gill et al. 2015; Greising et al. 2015b). In a previous study in mice, we found that DIAm sarcopenia is associated with an age‐related reduction in Pdi_max_ as well as Pdi generated during maximal ventilatory efforts against an occluded airway (Greising et al. 2015b). The Pdi generated during other ventilatory behaviors of the DIAm is unaffected in older mice.

In mice, we found that there are no sex‐related differences in body weight at 24 months of age when sarcopenia is observed in both males and females (Greising et al. 2015b). In rat models of aging, including Fischer 344 (F344) rats, there are sex‐related differences in body weight at 24 months of age reflecting differences in weight gain across their lifespan (Cameron et al. 1985; Rao et al. 1990; Kwekel et al. 2010). We previously observed DIAm sarcopenia in male F344 rats (Elliott et al. 2016c), but female animals were not included in this study. As body weight continues to increase in older female F344 rats while it plateaus in male rats (Cameron et al. 1985; Rao et al. 1990; Turturro et al. 1999; Kwekel et al. 2010), differences in DIAm sarcopenia may exist. Accordingly, we hypothesized that in F344 rats, there are sex‐related differences in DIAm sarcopenia and its impact on Pdi generation across DIAm motor behaviors.

## Materials and Methods

### Animals

All protocols were approved by the Mayo Clinic Institutional Animal Care and Use Committee. Fischer 344 rats acquired from the National Institute of Aging colony (Bethesda, MD) were used for this study. DIAm force was evaluated in 6‐month‐old (*n* = 8 males, *n* = 10 females) and 24‐month‐old (*n* = 8 males, *n* = 10 females) rats. Pdi generation was measured across a range of motor behaviors in 6‐month‐old (*n* = 9 male, *n* = 8 female) and 24‐month‐old (*n* = 9 male, *n* = 9 female) rats. In a subset of 12 rats (2–4 per age per sex) Pdi was measured followed by DIAm force experiments. There was no evidence of a difference in Pdi generation or force between this subset and rats that were used exclusively for either Pdi or DIAm force experiments; as such, data from these animals were combined with the accompanying individually tested groups. Muscle strips from the side of the diaphragm not tested during isometric DIAm force measurements were excised for cross‐sectional area measurements in a subset of 6‐month‐old (*n* = 6 males, *n* = 6 females) and 24‐month‐old (*n* = 7 males, *n* = 8 females) rats. A power analysis was performed by assuming similar variance in our outcome measures as in the same measures in previous studies in F344 rats (Gosselin et al. 1994; Elliott et al. 2016c) and a biologically relevant effect size of 15%. Animals were maintained on an alternating 12:12 h light‐dark cycle and had ad libitum access to fresh water and rat chow. An acclimation period of at least 48 h was provided before conducting any experimental procedures, and animals were weighed weekly until the terminal experiment was performed. Animals were anesthetized by intramuscular injection of ketamine (100 mg kg^−1^, JHP Pharmaceuticals, Rochester, MI) and xylazine (10 mg kg^−1^, MWI Veterinary Supply Co, Boise, ID), and anesthetic depth was confirmed by the absence of palpebral reflex and response to toe pinch prior to commencing the experiment.

### Diaphragm muscle isometric force

Methods for assessment of DIAm isometric force generation have been previously described (Lewis et al. 1986; Holter et al. 1987; Sieck et al. 1989b; Lewis et al. 1992a,b; Gosselin et al. 1996; Elliott et al. 2016c). Briefly, a DIAm strip ~2 mm wide was excised and positioned in a double‐walled glass chamber containing Reese‐Simpson buffer maintained at 26°C and aerated with 95% O_2_, 5% CO_2_ gas. The DIAm strip was secured by clamping the rib segment to a micropositioner and tying the central tendon to a force transducer (Model 6350, Cambridge Technology, Cambridge, MA) using 6–0 silk suture (Ethicon, New Jersey). The DIAm strip was electrically stimulated via platinum plate electrodes positioned on both sides of the muscle using square‐wave pulses of 2 msec duration (Grass S88; SIU5D Stimulus Isolation Unit; Grass Telefactor, Warwick, RI). Fiber length was increased until the optimal length (*L*_0_) for maximum isometric twitch force generation was observed. Stimulation current was increased until a peak twitch force (*P*_t_) was achieved and then set at 125% of this current strength (supramaximal stimulation, ~150 mA). The DIAm strip was then stimulated across a range of frequencies (5, 10, 20, 30, 40, 50, 75, and 100 Hz) in 1 sec duration trains with rest periods of 2 min between each frequency to assess the force–frequency response and to determine the maximal tetanic force (*P*_0_). Following force measurements, the length of the DIAm strip was determined using a digital caliper and muscle weight was determined after blotting away moisture and discarding the rib and central tendon tissue. Physiological cross‐sectional area was estimated by the quotient of muscle weight and *L*_0_ x muscle‐specific density (1.056 g cm^−3^). Specific force was determined by normalizing forces for DIAm cross‐sectional area and expressed as *N* cm^−2^.

### Transdiaphragmatic pressure measurements

The Pdi was calculated as the difference between the gastric and esophageal pressures as previously described (Watchko et al. 1986; Sieck and Fournier 1989; Mantilla et al. 2010; Greising et al. 2013; Gill et al. 2015; Greising et al. 2016; Khurram et al. 2017). Briefly, in spontaneously breathing anesthetized rats, two 3.5 French Millar solid‐state pressure catheters (SPR‐524; Millar Instruments, Houston, TX) were inserted into the esophagus and the stomach, spanning the thoracic and abdominal borders of the DIAm. During eupneic breathing, correct catheter position was confirmed based on appropriate direction of signal deflection of the gastric and esophageal pressures. The abdomen was bound for the duration of the pressure measurements and tension of the binding was adjusted to optimize both the gastric and esophageal pressures, thus approximating isometric conditions and minimizing changes in pulmonary functional residual capacity.

Pdi measurements were obtained during: (1) quiet breathing of room air (eupnea), (2) breathing stimulated by exposure to a hypoxic–hypercapnic (10% O_2_–5% CO_2_) gas mixture for 5 min, (3) inspiratory efforts against an occluded airway, and (4) bilateral phrenic nerve stimulation at 100 Hz (Pdi_max_). Deep breaths (“sighs”) were defined as spontaneously occurring larger (~2× normal eupneic Pdi) inspiratory events with a biphasic Pdi trajectory (Mantilla et al. 2010; Li et al. 2016) followed by apnea with elimination of at least one full breath. The animals were rested for at least 2 min between behaviors to allow Pdi amplitude to return to eupneic baseline.

During airway occlusion measurements, the mouth and nose of the rat were completely covered by a standard latex glove and enough external pressure was applied to obstruct airflow for approximately 45 sec. Typically, during this time, the animal made ~30 strained efforts of increasing amplitude until a plateau was reached. Following a rest period after airway occlusion, surgical microdissection was performed in the ventral cervical region to bilaterally expose and isolate phrenic nerves prior to stimulation. Straight parallel bipolar electrodes (125 *μ*m platinum iridium wires with 0.5‐mm interelectrode spacing; FHC, #PBSD0875, Bowdoin, ME) were used to stimulate (Grass S88 stimulator) the phrenic nerves (0.05 msec duration ~10 mA pulses at a frequency of 100 Hz in a 330 msec duration train). To avoid current spread, a mineral oil bath was created in the neck surrounding the isolated phrenic nerve. During supramaximal stimulation, deflections in esophageal and gastric pressures were monitored and verified to be in the appropriate directions. If inappropriate, placement of the pressure transducers was adjusted. In addition, we verified that the duration of the Pdi response matched the stimulation period, and that there were no obvious signs of movement artifact in the individual esophageal and gastric signals or the resulting Pdi signal.

Esophageal and gastric pressures were measured independently, digitized (400 Hz) with PowerLab 4/35 and visualized using LabChart 8 (ADInstruments, Colorado Springs, CO). The Pdi signals were band‐pass filtered between 0.3 and 30 Hz. Data were exported to MATLAB (MathWorks, Natick, MA), down sampled to 100 Hz, and analyzed post hoc using a custom‐designed script for semi‐automated detection of Pdi amplitude and ventilatory parameters (Medina‐Martinez et al. 2015). Behaviors were analyzed for variable periods, with all spontaneous deep breaths evaluated, a 1–2 min duration period sampled for eupnea, the final 2 min evaluated for hypoxia–hypercapnia, 2–5 maximal breaths evaluated during occlusion, and 1–5 maximal stimulation events analyzed for bilateral phrenic nerve stimulation. Results from one animal were excluded because Pdi amplitude during eupnea was very low – 2 standard deviations outside the mean value derived from aggregated data in previously published studies (Mantilla et al. 2010; Gill et al. 2015; Khurram et al. 2017).

### Fiber‐type cross‐sectional areas

Prior to flash freezing DIAm strips were stretched to ~150% resting length, which approximates the *L*_0_ measured during the measurement of DIAm *P*_0_. Serial 10 *μ*m transverse sections were cut using a Reichert Jung Frigocut 2800 Cryostate (Reichert Microscope Services, Depew, NY) from fresh‐frozen DIAm samples. Sections were fixed in acetone for 10 min prior to commencing immunofluorescence staining protocols. Sections were blocked for 30 min in 10% goat serum and incubated overnight at 4°C in primary antibodies for the following MyHC isoforms: MyHC_Slow_ (BA‐F8, 1:3 dilution; Developmental Studies Hybridoma Bank, Iowa City, IA), MyHC_2A_ (SC‐71, 1:3 dilution; Developmental Studies Hybridoma Bank), and Laminin (Sigma L9393, 1:200 dilution; Sigma‐Aldrich, St. Louis, MO). Fluorescently conjugated secondary antibodies were then applied at a 1:200 dilution, using Alexa Fluor 488 to visualize MyHC_Slow_ and Alexa Fluor 568 to visualize MyHC_2A_ and Alexa Fluor 405 to visualize laminin. Based on the staining pattern, DIAm fibers were classified as Type I, Type IIa, and Type IIx and/or IIb (Sieck et al. 1989a; Prakash and Sieck 1998; Greising et al. 2013; Elliott et al. 2016c).

Muscle cross‐sections were imaged using a 20× oil‐immersion objective (NA 1.0) on an Olympus FV2000 laser confocal microscope (Olympus America, Melville, NY) capable of simultaneous multilabel fluorescence imaging. Images were captured in a 1200 × 1200 pixel array, with similar acquisition parameters across preparations. Proportions and cross‐sectional area (CSA) of DIAm fiber types as well as the interstitial space (including vascular areas) were determined using morphometric tools in ImageJ.

### Statistics

All statistical evaluation was performed using standard statistical software (JMP Pro 11, SAS Institute Inc., Cary, NC), with data assessed for normality using D'Agostino and Pearson omnibus tests, with exclusion based on a priori criteria of Pdi amplitude or isometric DIAm force measurements being outside a range of two times the standard deviation away from the mean. Differences in DIAm force were evaluated using a two‐way repeated measures analysis of variance with age and sex as the main effects. Differences in Pdi and ventilatory parameters were evaluated using a three‐way analysis of variance with age, sex, and behavior as the main effects. Both sexes were powered to be able to detect a 15% difference in Pdi during bilateral phrenic nerve stimulation. When appropriate, post hoc analyses were conducted using the Tukey–Kramer Honestly Significant Difference test. Statistical significance was established at *P *<* *0.05. All experimental data in the text of the manuscript are presented as mean ± 95% confidence interval across animals, unless otherwise specified.

## Results

### Animals

Among the 24‐month‐old F344 rats, ~20% were excluded due to the presence of a tumor detected upon necropsy or a failure to thrive after the animals arrived. There was no apparent sex difference in the exclusion of these rats even though the survival rate of F344 rats at 24 months is 50% in males and closer to 80% in females (Turturro et al. 1999). The body weight of males (Young: 365 ± 5 g, Old: 400 ± 7 g) was significantly higher than that of females (Young: 186 ± 2 g, Old: 252 ± 7 g) in both age groups at the time of the experiment. The weight of aged rats of both sexes was significantly higher than their younger counterparts (*P *<* *0.01). In female animals there was a ~35% increase in body weight from 6 to 24 months of age, compared to a more modest ~10% increase in male animals.

### Diaphragm muscle isometric force

**Figure 1:**
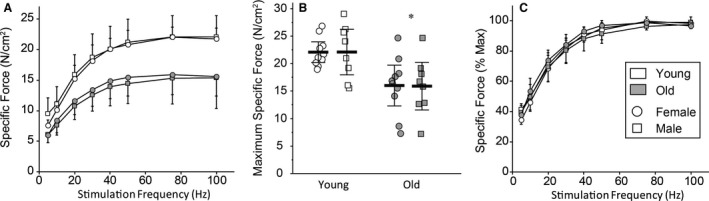
Diaphragm muscle force–frequency curves (A) and maximal specific force, *P*_0_ (B), from young and old Fischer 344 rats show a ~30% age‐related reduction in *P*_0_ in both sexes. Force normalized to the maximal force (C) shows no evidence of a shift between young and old animals of either sex. *Significantly different from young rats for both sexes. Data are shown as means ± 95% CI.

Force–frequency curves are plotted in Figure 1 for males and females in young and old animals. There was a main effect on specific force of age (*F*_1,17_ = 17, *P *<* *0.01) and frequency (*F*_7,31_ = 79, *P *<* *0.01), but not sex (*F*_1,37_ < 1, *P* = 0.97). Post hoc analysis revealed no evidence for a difference between males and females or young and old animals at any stimulation frequency. However, there was a main effect on *P*_0_ of age (*F*_1,39_ = 19, *P *<* *0.01), but not sex (*F*_1,39_ < 1, *P* = 0.76) or the age*sex interaction (*F*_1,39_ < 1, *P* = 0.99). Post hoc analysis revealed no evidence of a sex‐related difference, but a stark ~30% reduction in *P*_0_ in aged rats of both sexes (Fig. 1). There was also a main effect on *P*_t_ of age (*P*_t_: *F*_1,39_ = 9; *P* < 0.01) and sex (*F*_1,39_ = 6; *P* = 0.02), but not their interaction (*F*_1,31_ ≤ 1; *P* = 0.88). Post hoc analysis revealed that there was no evidence for a difference in *P*_t_ between males and females or young and old animals. There was a main effect on the *P*_t_:*P*_0_ ratio of sex (*F*_1,39_ = 14, *P* < 0.01), but not age or the age*sex interaction. Post hoc analysis revealed that females display ~80% of the *P*_t_:*P*_0_ ratios of males, reflecting the lower *P*_t_ in younger females (Table 1).

**Table 1:** Values are means ± 95% CI

Age	Young (6 months)		Old (24 months)	
Sex	Female	Male	Female	Male
Body weight (g)	187 ± 4	371 ± 23^b^	244 ± 10^a^	405 ± 20^a^,^b^
*L*_0_ (cm)	2.10 ± 0.12	2.29 ± 0.14	2.27 ± 23	2.68 ± 0.17
*P*_t_/CSA (N cm^−2^)	6.9 ± 0.8	8.7 ± 2.1^b^	5.5 ± 1.1^a^	5.6 ± 1.3^a^
*P*_0_/CSA (N cm^−2^)	22.1 ± 1.6	22.1 ± 3.4	16.0 ± 3.2^a^	15.9 ± 3.6^a^
*P*_t_/*P*_0_	0.31 ± 0.02	0.38 ± 0.04^b^	0.35 ± 0.04	0.36 ± 0.05
Interstitial space (%)	27 ± 2	22 ± 8	26 ± 6	21 ± 9

*L*_0_, optimal diaphragm muscle segment length; *P*_0_, maximal tetanic tension; *P*_t_, peak twitch force; *P*_t_/*P*_0_, peak twitch force to maximal tetanic tension ratio; CSA, cross‐sectional area.

a Significantly different from young.

b significantly different from female.

### Diaphragm muscle fiber‐type proportions, cross‐sectional areas, and relative contributions

**Figure 2:**
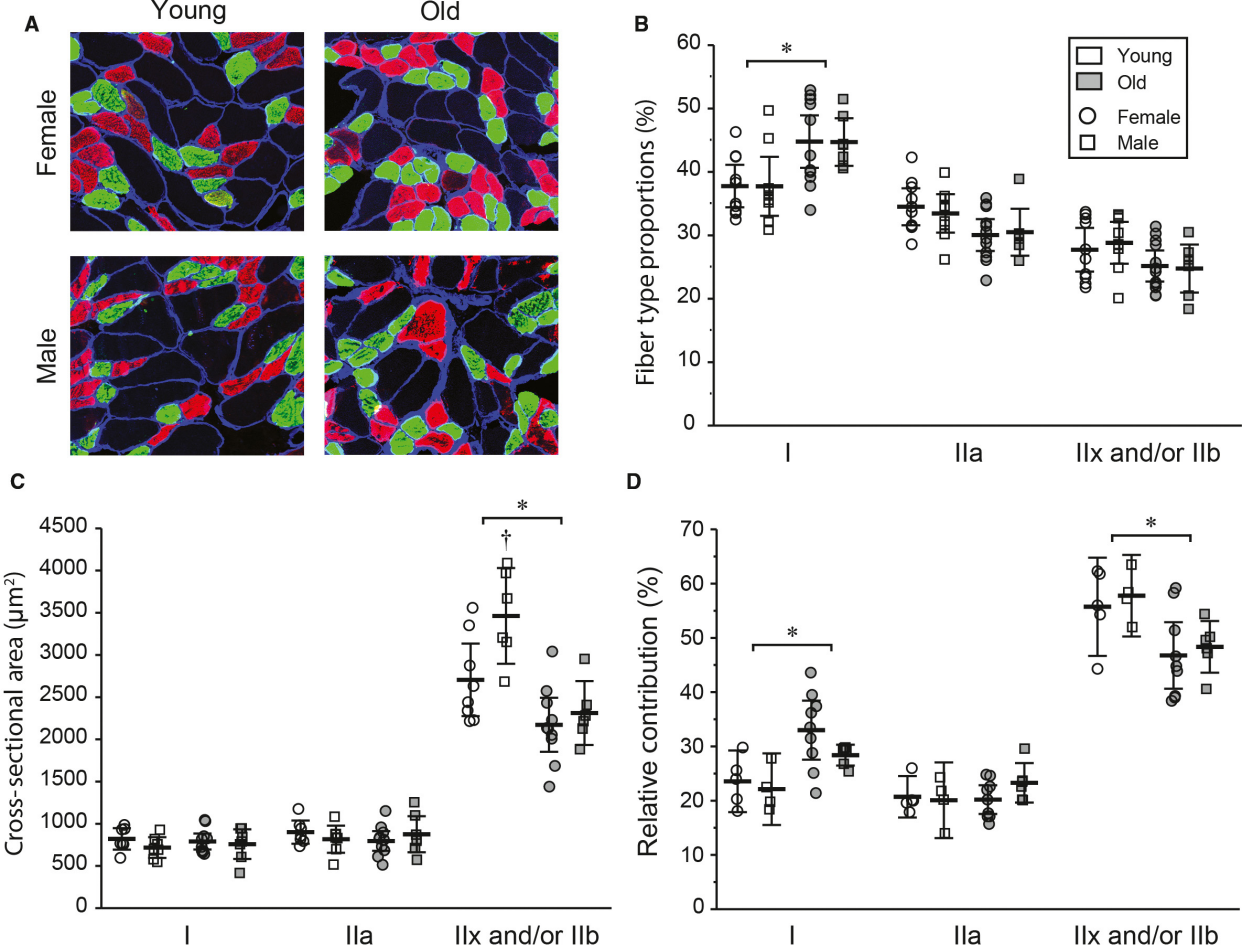
Representative diaphragm muscle cross‐sections (A), fiber‐type proportion (B), average cross‐sectional areas for muscle fiber types (C), and relative contributions (D) from young and old Fischer 344 rats of both sexes. Data points are average cross‐sectional area from each animal, while the summarized data (black line) are the mean ± 95% CI across animals. *, Significantly different from young rats; †, significantly different from females within the same age group.

The proportions of DIAm fiber types in young and old F344 rats of both sexes are shown in Figure 2. There were changes in DIAm fiber‐type proportions that depended on fiber type (*F*_2, 102_ = 92, *P* < 0.01) and interacted with age (age*fiber‐type interaction; *F*_2,102_ = 16, *P* < 0.01), but not sex. In young animals, there were no differences in the proportions of type I and IIa DIAm fibers, which were both significantly more prevalent than type IIx and/or IIb fibers (*P* < 0.01). By 24 months of age, the proportion of type I muscle fibers significantly increased by ~20% while the proportion of type IIx and/or IIb DIAm fibers tended to be reduced.

Average CSA's of different DIAm fiber types in young and old animals of both sexes are shown in Figure 2. There was a main effect on DIAm fiber CSA of age (*F*_1,69_ = 35, *P* < 0.01), sex (*F*_1,69_ = 7, *P* < 0.01), and fiber type (*F*_2,69_ = 394, *P* < 0.01). There was no evidence for a difference in CSA's of type I and type IIa muscle fibers within or between all intersections of age and sex (young males, young females, old males, and old females). Furthermore, in all intersections of age and sex, the cross‐sectional area of type IIx and/or IIb muscle fibers was larger than the cross‐sectional area of type I and type IIa muscle fibers. In addition, although there was no evidence of a difference in the cross‐sectional area of type I and type IIa muscle fibers between males and females at either age, young males had larger type IIx and/or IIb muscle fiber CSA's than young females. By 24 months of age, the evidence for a difference in type IIx and/or IIb muscle fiber CSA's between sexes was no longer present. Importantly, in both males and in females, the CSA of type IIx and/or IIb muscle fibers was reduced by ~30% in old age. There was no evidence for a difference in the percentage of total muscle CSA comprising interstitial space among sex (*F*_1,21_ = 2 *P *≥* *0.22), age (*F*_1,21_ < 1, *P* = 0.96), or their interaction (*F*_1,21_ < 1, *P* = 0.95). Data are reported in Table 1.

The relative contribution of DIAm fiber types was defined as the product of the fiber‐type proportion and average CSA of each fiber type (Fig. 2). There were differences in the relative contributions of fiber types (*F*_2,60_ = 213, *P* < 0.01) that interacted with age (age*fiber‐type interaction; *F*_2,60_ = 14, *P* < 0.01), but not sex. The relative contribution of type I DIAm fibers increased by 30% in older animals, while the relative contribution of type IIx and/or IIb fibers was reduced by ~20%.

### Transdiaphragmatic pressure across diaphragm motor behaviors

**Figure 3:**
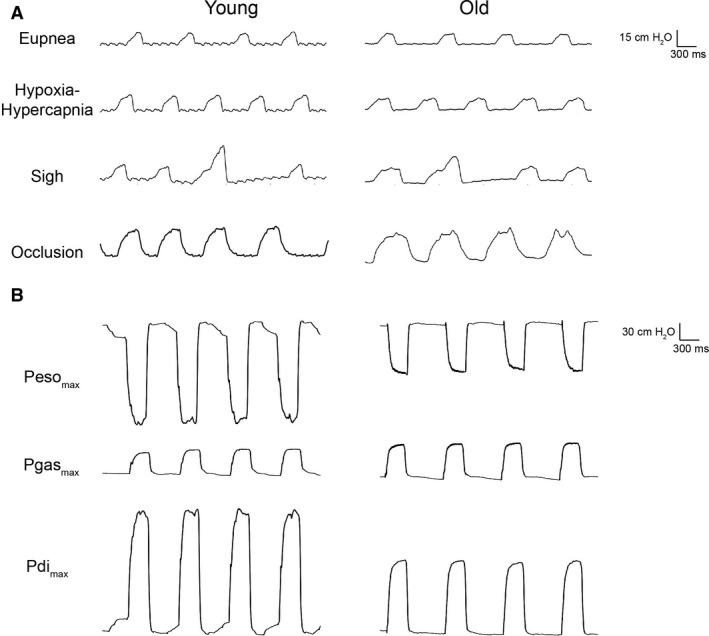
Representative transdiaphragmatic pressure tracings across motor behaviors (A) and during bilateral phrenic nerve stimulation (B). Esophageal and gastric pressure tracings during phrenic nerve stimulation are also shown, and were verified to deflect in the appropriate direction for all behaviors.

**Figure 4:**
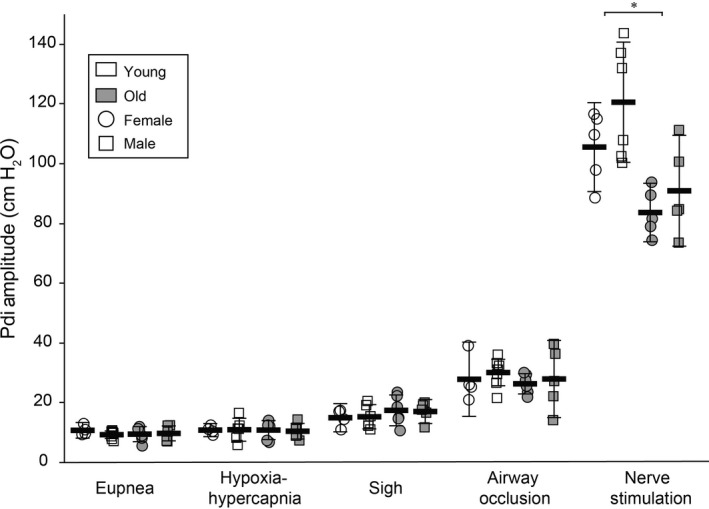
Transdiaphragmatic pressure generation across motor behaviors and during bilateral phrenic nerve stimulations in young (open) and old (gray) animals of both sexes (circle – female; square – male). There was no evidence of impairment in Pdi generation during ventilatory behaviors (eupnea, hypoxia–hypercapnia, sigh, and airway occlusion). By contrast, there was ~20% reduction in Pdi_max_ (nerve stimulation) in old age in both sexes. *, Significantly different from young rats for both sexes. Data are shown as mean ± 95% CI.

Representative Pdi tracings from a single young and a single old animal are shown in Figure 3. Average data from individual animals of both sexes at each age across DIAm motor behaviors requiring varying levels of force are shown in Figure 4. There was a main effect on Pdi amplitude of age (*F*_1,83_ = 45, *P* < 0.01) and behavior (*F*_4,83_ = 402, *P* < 0.01), but not sex (*F*_1,83_ < 1, *P* = 0.73). The age*behavior interaction was also significant (*F*_4,83 _= 12, *P* < 0.01), but the age*sex (*P* = 0.59), sex*behavior (*P* = 0.06), and age*sex*behavior (*P *=* *0.87) interactions were not. Post hoc analysis revealed no significant differences in Pdi amplitude between young and old rats of either sex during eupnea (Young: 10 ± 1 cm H_2_O, *n* = 11; Old: 10 ± 2 cm H_2_O, *n* = 12), hypoxia–hypercapnia (Young: 11 ± 2 cm H_2_O, *n* = 10; Old: 11 ± 2 cm H_2_O, *n* = 12), sighs (Young: 15 ± 2 cm H_2_O; Old: 17 ± 3 cm H_2_O), or airway occlusion (Young: 29 ± 3 cm H_2_O, *n* = 11; Old: 27 ± 4 cm H_2_O, *n* = 11). However, the combined male and female Pdi_max_ was significantly reduced by ~20% in older compared to younger animals (Young: 112 ± 10 cm H_2_O, *n* = 11; Old: 89 ± 10 cm H_2_O, *n* = 11; *P* < 0.01).

### Ventilatory parameters

Ventilatory parameters were determined during eupnea and hypoxia–hypercapnia and details are provided in Table 2. There was a main effect on respiratory rate of age (*F*_1,37_ = 10, *P *<* *0.01) and behavior (*F*_1,37_ = 16, *P *<* *0.01), but not sex (*F*_1,37_ < 1, *P* = 0.42). A significant effect on the age*sex (*F*_1,37_ = 12, *P *<* *0.01) and age*behavior*sex (*F*_1,37_ = 7, *P* = 0.01), but not the age*behavior or behavior*sex interactions was also observed. Overall, respiratory rate in young and old animals during eupnea was 53 ± 6 min^−1^ and 44 ± 7 min^−1^, respectively. During hypoxia–hypercapnia, respiratory rate in young and old animals was 77 ± 12 min^−1^ and 58 ± 10 min^−1^, respectively. Post hoc analysis also revealed that in young rats, hypoxia–hypercapnia elicited a significantly larger increase in respiratory rate in males (from 55 ± 4 min^−1^ during eupnea to 89 ± 6 min^−1^) compared to females (from 50 ± 5 min^−1^ during eupnea to 60 ± 6 min^−1^). No such sex differences were present in older rats.

**Table 2:** Ventilatory parameters in anesthetized young and old Fischer 344 rats of both sexes

Age	Sex	Eupnea			Hypoxia–Hypercapnia		
		Respiratory rate (min^−1^)	Inspiratory duration (msec)	Duty cycle (%)	Respiratory rate (min^−1^)	Inspiratory duration (msec)	Duty cycle (%)
Young (6 months)	Female	50 ± 10	392 ± 60	32 ± 3	60 ± 13	375 ± 31	37 ± 2
	Male	55 ± 9	385 ± 60	33 ± 8	89 ± 11^a^,^b^	308 ± 28	45 ± 9
Old (24 months)	Female	45 ± 8	438 ± 72	31 ± 6	48 ± 7	381 ± 66	44 ± 16
	Male	43 ± 13	412 ± 45	29 ± 7	68 ± 15	437 ± 71	35 ± 8

a Significantly different from young.

b Significantly different from female.

There was a main effect on duty cycle of behavior (*F*_1,37_ = 7, *P* = 0.01), but not age (*F*_1,37_ < 1, *P* = 0.90), sex (*F*_1,37_ < 1, *P* = 0.97), or any of the interactions. During eupnea, the duty cycle was 33 ± 4% in young animals and 30 ± 4% in old animals. During hypoxia–hypercapnia, the duty cycle increased in young animals to 41 ± 6% and in old animals to 40 ± 9%.

There was no evidence for a main effect on inspiratory duration of age (*F*_1,37_ = 4 *P* = 0.05), behavior (*F*_1,37_ = 1, *P* = 0.28), sex (*F*_1,37_ < 1, *P* = 0.88), or any of the interactions. The inspiratory duration during eupnea was 388 ± 40 msec in young and 413 ± 41 msec in old animals. During hypoxia–hypercapnia, inspiratory duration was 342 ± 40 msec and 410 ± 49 msec in young and old animals, respectively, although none of these differences were significant.

## Discussion

The results of this study demonstrate that DIAm sarcopenia is present in 24‐month‐old F344 rats of both sexes. Indeed, in old animals, both *P*_0_ and the CSA of type IIx and/or IIb DIAm fibers were reduced by ~30% regardless of sex. These findings are consistent with previous observations in male F344 rats (Gosselin et al. 1994; Elliott et al. 2016c), but this study extends these observations to female F344 rats and provides a functional measure of the ability of the DIAm to accomplish a range of motor behaviors, that is, Pdi. In this study, Pdi_max_ was reduced by ~20%, suggesting that near‐maximal airway clearance behaviors necessary to maintain airway patency may be impaired. Reduced force production of the DIAm may also impair other nonventilatory expulsive motor behaviors requiring near‐maximal DIAm activation. These behaviors require the generation of large, positive, intra‐abdominal pressures and include Valsalva straining maneuvers (Cobb et al. 2005; Hackett and Chow 2013; Shaw et al. 2014), vomiting (Abe et al. 1993, 1994), defecation (Fukuda et al. 1981; Fukuda and Fukai 1986b,a, 1988; Carry and Banssillon 1994), and parturition (Higuchi et al. 1987). The presence of sarcopenia clearly results in reduced DIAm functional capacity in both male and female rats, as evidenced by the reduction in Pdi_max_ and DIAm *P*_0_. The findings of this study do not support our hypothesis that there are sex‐related differences in the manifestation of sarcopenia (i.e., muscle weakness and atrophy) in F344 rats.

Respiratory muscles including the DIAm are important for sustaining ventilation and are thus highly active throughout life. Additionally, these muscles are important during higher‐force behaviors and near‐maximal expulsive behaviors necessary for airway clearance (Sieck 1988, 1989; Sieck and Fournier 1989; Mantilla and Sieck 2011; Mantilla et al. 2014a) and during nonventilatory behaviors that necessitate increased abdominal pressure. The results of this study demonstrate that the Pdi generated during ventilatory behaviors, including inspiratory efforts against a closed airway, is not impaired in old age. Given the large reserve capacity of the DIAm and the relatively low pressures generated during these behaviors (<50% Pdi_max_), these behaviors could likely be accomplished by recruitment of lower‐threshold type S and FR motor units alone (Sieck and Fournier 1989; Mantilla et al. 2010). The lack of evidence of a functional impact of sarcopenia on ventilatory behaviors is not at all surprising given that even after hemidiaphragm paralysis induced by unilateral phrenicotomy, Pdi generation during ventilatory behaviors remains unimpaired (Gill et al. 2015), especially over time after injury (Khurram et al. 2017). The response to airway occlusion in F344 rats represents the maximal inspiratory effort possible against a closed airway. In this study, Pdi generated during the response to airway occlusion was not impaired with old age.

We recently showed that compared to younger rats, in 24‐month‐old F344 rats, there are approximately 50% fewer phrenic motor neurons in the upper tertile of the motor neuron surface area distribution, which corresponds to a 20% reduction in the overall number of phrenic motor neurons (Fogarty et al. 2018b). This profile of motor neuron loss is entirely consistent with the impairment of near‐maximal DIAm motor behaviors that require recruitment of type FInt and FF motor units (Sieck 1988, 1989; Sieck and Fournier 1989; Mantilla and Sieck 2011; Mantilla et al. 2014a), as well as the selective reduction in type IIx and/or IIb DIAm fibers. Previously, we showed that denervation results in the selective atrophy of type IIx and/or IIb DIAm fibers (Miyata et al. 1995; Geiger et al. 2001), and thus may underlie the selective atrophy of these DIAm fibers in old age reported in this study as well as previous studies (Greising et al. 2013; Elliott et al. 2016c). We previously reported that DIAm P_0_ is reduced in aged mice of both sexes (Greising et al. 2015b) and male F344 rats (Elliott et al. 2016c). Regardless of sex, P_0_ in this study was reduced in old animals by ~30%, generally consistent with previous reports (Gosselin et al. 1994; Elliott et al. 2016c).

It is likely that the vulnerability of type FInt and FF motor units in aging is related to both neurogenic and myogenic factors. Sadly, the exact mechanisms that contribute to the selective denervation of type FInt and FF motor units remain unknown. However, aging studies that have given credence to motor unit type, along with lesion studies (unilateral DIAm denervation) and lessons from neurodegenerative conditions (amyotrophic lateral sclerosis, ALS) that selectively afflict type FInt and FF motor units, provide some insight into possible mechanisms of selective vulnerability.

Unilateral denervation of the DIAm results in the selective atrophy of type IIx and/or IIb muscle fibers (Miyata et al. 1995; Zhan et al. 1995; Geiger et al. 2001; Aravamudan et al. 2006; Sieck et al. 2007; Argadine et al. 2009). Importantly, after denervation the specific force of type IIx and/or IIb single muscle fibers is reduced to the level of type I muscle fibers, which remain unaffected (Geiger et al. 2001). Functional assessment of Pdi following unilateral DIAm denervation shows preserved ventilatory behaviors and impaired higher‐force behaviors (Gill et al. 2015; Khurram et al. 2017). Future studies examining changes in myosin heavy‐chain protein content and how this affects the specific force of single muscle fibers (Geiger et al. 1999, 2000) from young and old rats will provide important insights into the mechanisms of motor unit type‐dependent changes.

Type FInt and FF motor units are selectively vulnerable in ALS (Kiernan and Hudson 1991; Dukkipati et al. 2018; Fogarty 2018), a condition afflicting corticospinal neurons and motor neurons (Fogarty et al. 2015, 2016a,b, 2017). The mechanisms underlying the loss of motor neurons and muscle‐specific force in ALS may be similar to those underlying motor neuron loss and muscle weakness during aging. In ALS, marked disruptions of neurotrophic signaling (including brain‐derived neurotrophic factor [BDNF] and its high‐affinity tropomyosin‐related kinase B [TrkB] receptor) are apparent, with increased neurotrophic support promoting motor neuron survival in ALS models (Henderson et al. 1994; Kishino et al. 1997; Das et al. 2016). During aging, the availability of TrkB receptors appears to decline in DIAm motor units (Greising et al. 2015b, 2017), although fiber‐type‐specific effects are currently unknown. Inhibiting TrkB signaling in young adult rodents results in neuromuscular transmission failure and neuromuscular junction defects at type IIx and/or IIb fibers (Mantilla et al. 2014b; Greising et al. 2015c), which are remarkably similar to the deficits observed during old age (Prakash and Sieck 1998; Valdez et al. 2012; Greising et al. 2015b). Mitochondrial dysfunction in ALS, with fragmentation of mitochondria preceding neuronal death (Kong and Xu 1998; Vande Velde et al. 2011; Vinsant et al. 2013), is concomitant with functional changes throughout the neuraxis (van Zundert et al. 2008; Fogarty et al. 2015; Jiang et al. 2017). The enhancement of mitochondrial fusion and/or the inhibition of mitochondrial fragmentation ameliorates neuronal degeneration in ALS models (Song et al. 2013; Wang et al. 2013). An intriguing association of neurotrophic signaling and the enhancement of mitochondrial fusion (Su et al. 2014) may be related to reports of TrkB receptors on the outer mitochondrial membranes (Wiedemann et al. 2006). Taken together, an examination of motor unit type‐specific changes in muscle fibers and motor neurons of neurotrophic signaling and mitochondria may provide useful insights into the mechanism of age‐related sarcopenia.

We previously showed that Pdi_max_ was reduced by ~27% in old mice (Greising et al. 2015b), which is similar to the ~20% age‐related reduction in Pdi_max_ in this study in F344 rats. Absolute Pdi (cm H_2_O) values recorded during all respiratory motor behaviors were similar in this study to those previously measured in our laboratory (Mantilla et al. 2010; Gill et al. 2015; Khurram et al. 2017). The Pdi_max_ in this study (~110 cm H_2_O) is similar to Pdi_max_ in mice (~75 cm H_2_O (Greising et al. 2013, 2015b), piglets (~80 cm H_2_O (Watchko et al. 1986; Mayock et al. 1990)), sheep (~75 cm H_2_O (Bazzy et al. 1988), and rabbits (~70 cm H_2_O (Ferguson et al. 1990)). With regard to differences Pdi_max_, species, lung volume, and stimulation technique may play a role. In this study, we were highly cognizant of detecting appropriate and opposite directions of deflection in both the esophageal and gastric pressures during bilateral nerve stimulation.

The Pdi_max_ in this study was reduced less (~20%) than what would be expected based on the 30% reduction in *P*_0_. Of note, Pdi_max_ in aged humans (as measured by the Mueller maneuver or the sniff test) is reduced by ~15–25% compared to healthy young adults (Tolep et al. 1995; Polkey et al. 1997). Additionally, in previous studies with F344 rats, DIAm *P*_0_ was reduced by closer to 20% (Gosselin et al. 1994; Elliott et al. 2016c). Thus, across multiple studies, the apparent discrepancy between Pdi_max_ and DIAm *P*_0_ seems to be minimal. However, it is possible that age‐related changes in the alveolar structure and/or lung and chest wall mechanics may be altering the measurement of the pressure signal, thus obscuring the extent of deficit in Pdi generation. In old age, alveolar airspace wall surface area per unit volume tends to decrease in humans (Gillooly and Lamb 1993), the combined lung and chest wall compliance may increase slightly (Elliott et al. 2016b) in mice or be unchanged in humans (Turner et al. 1968), and although the residual volume in the lung is slightly increased in older humans (Turner et al. 1968; Janssens et al. 1999), functional residual capacity is either unchanged or increases less than the concomitant change in residual volume (Turner et al. 1968; Tolep et al. 1995; Janssens et al. 1999). Regardless, it is evident that these changes in lung and chest wall mechanics associated with old age would be more likely to result in an increase in the radius of curvature of the DIAm, leading to a greater‐than‐expected reduction in the measured pressure. Thus, age‐related changes in lung and chest wall mechanics are unlikely to explain this possibility. The most likely scenario is simply that the reduction in DIAm *P*_0_ may have been at the higher end of the expected 20–30% reduction in this study, partly due to the high variability in the measurement of 24‐month‐old rats. It remains unclear why these measurements were more variable (CV ~30%) in this study than in previous studies, however, the mean P_0_ values are similar to previous studies for both young and old rats (Gosselin et al. 1994; Elliott et al. 2016c). We also investigated whether the variability in *P*_0_ was related to differences in CSA across animals. As the CSA of type IIx and/or type IIb muscle fibers was reduced in old age, we performed a Pearson regression analysis of type IIx and/or IIb muscle fiber CSA and *P*_0_. We found no evidence to suggest that P_0_ was highly correlated with the CSA (*R* ^2^ < 0.15 with a slope of ~0.1) in young, old, or the combined young and old groups.

Importantly, the absence of evidence for sex‐related differences at 6 and 24 months of age does not preclude potential sex differences at some age in between. Considering the sex‐related differences in average body weight changes over the age of the F344 rat (Cameron et al. 1985; Rao et al. 1990; Turturro et al. 1999; Kwekel et al. 2010), differences in DIAm function may exist at certain ages. In fact, previous studies have reported sex differences in DIAm protein content and SDH activity of young adult (12 months) Sprague–Dawley rats (Lawler et al. 1994) and nutritional deprivation‐induced changes in DIAm fiber cross‐sectional area in Wistar rats (Prezant et al. 1994). Estrogen has direct effects on feeding behaviors in rats (Sieck et al. 1977, 1978), and the plateauing of weights in old age may partly reflect changes in sex hormones. We selected 24‐month‐old F344 rats based on the survival statistics (~50%) and the females being postmenopausal, thus minimizing differential sex hormone effects between males and females. The lack of evidence for a difference in sarcopenia between male and female rats is in good agreement with previous data in mice (Greising et al. 2013).

The results of this study demonstrate that sarcopenia is present in both male and female F344 rats at 24 months of age and results in a deficit in maximal Pdi. This reduction in DIAm force production is likely related to the loss of larger motor neurons (Fogarty et al. 2018b) as well as the selective atrophy of type IIx and/or IIb muscle fibers reported in this study as well as previous studies (Greising et al. 2013; Elliott et al. 2016c). The loss of larger phrenic motor neurons results in the denervation and subsequent atrophy of the type IIx and IIb muscle fibers. In this study, as well as in a previous study (Elliott et al. 2016c), we show an age‐related increase in the proportion of type I DIAm fibers. This change in fiber‐type proportions may serve a protective role to preserve lower‐force ventilatory behaviors. However, the combined effects of selective muscle fiber atrophy and phrenic motor neuron loss result in impairment of near‐maximal expulsive airway clearance behaviors and may underlie the increased risk for respiratory complications observed in the elderly (Sharma and Goodwin 2006; Lowery et al. 2013). Understanding the timeline of motor unit degeneration and sarcopenia is important in determining appropriate therapeutic windows that may inform approaches to mitigate their deleterious functional impact.

## Conflict of Interest

The authors have no conflicts, real or perceived, to declare.
